# Asymptomatic Rectal Bacterial Pathogens Show Large Prospective Relationships With HIV Incidence in a Cohort of Young Sexual and Gender Minorities: Implications for STI Screening and HIV Prevention

**DOI:** 10.1093/ofid/ofae444

**Published:** 2024-08-05

**Authors:** Ross A Baiers, Daniel T Ryan, Antonia Clifford, Erik Munson, Richard D’Aquila, Michael E Newcomb, Brian Mustanski

**Affiliations:** Institute for Sexual and Gender Minority Health and Wellbeing, Northwestern University, Chicago, Illinois, USA; Institute for Sexual and Gender Minority Health and Wellbeing, Northwestern University, Chicago, Illinois, USA; Institute for Sexual and Gender Minority Health and Wellbeing, Northwestern University, Chicago, Illinois, USA; Medical Laboratory Science, College of Health Sciences, Marquette University, Milwaukee, Wisconsin, USA; Department of Medicine-Infectious Diseases, Feinberg School of Medicine, Northwestern University, Chicago, Illinois, USA; Institute for Sexual and Gender Minority Health and Wellbeing, Northwestern University, Chicago, Illinois, USA; Department of Medical Social Sciences, Feinberg School of Medicine, Northwestern University, Chicago, Illinois, USA; Institute for Sexual and Gender Minority Health and Wellbeing, Northwestern University, Chicago, Illinois, USA; Department of Medical Social Sciences, Feinberg School of Medicine, Northwestern University, Chicago, Illinois, USA

**Keywords:** asymptomatic sexually transmitted infection, men who have sex with men, HIV incidence, HIV risk, sexually transmitted diseases

## Abstract

**Background:**

We estimated the predictive value of rectal (bacterial sexually transmitted infection [bSTI]) pathogen detection for future HIV seroconversion among young adult sexual and gender minorities (YSGMs) assigned male at birth (AMAB).

**Methods:**

Data were collected between March 2018 and August 2022 from RADAR, a longitudinal cohort study of YSGMs AMAB living in the Chicago metropolitan area (n = 1022). Rates of rectal bSTIs and the proportion of self-reported rectal bSTI symptoms are reported. We examined whether the presence of rectal bSTIs predicted HIV seroconversion using generalized estimating equations (GEEs).

**Results:**

Participants tested reactive for rectal *Mycoplasma genitalium* (MGen), *Neisseria gonorrhoeae* (NG), and *Chlamydia trachomatis* (CT) at a rate of 20.8 (95% CI, 18.4–23.5), 6.5 (95% CI, 5.0–8.2), and 8.4 (95% CI, 6.8–10.3) cases per 100 persons, respectively. There were no statistically significant pairwise differences in self-reported rectal bSTI symptoms between participants with self-collected swabs testing nonreactive vs reactive for rectal MGen (χ^2^ = 0.04; *P* = .84), NG (χ^2^ = 0.45; *P* = .37), or CT (χ^2^ = 0.39; *P* = .46). In multivariate GEE analysis, rectal NG (adjusted odds ratio, 5.11; 95% CI, 1.20–21.77) was a statistically significant predictor of HIV seroconversion after controlling for other bSTIs, demographics, and sexual risk behavior.

**Conclusions:**

Our findings provide a robust longitudinal estimation of the relationship between primarily asymptomatic rectal NG nucleic acid detection and HIV infection. These findings highlight the importance of asymptomatic screening for bSTIs and targeting biobehavioral intervention to prevent HIV infection among YSGMs with rectal bSTI agents detected.

Sexually transmitted infections (STIs) have been on the rise in the United States for decades, particularly among young adult sexual and gender minorities (YSGMs) assigned male at birth [1]. In 2020, 53% of the 2.4 million new cases of STIs were found in young adults age 15–24 years. Men who have sex with men (MSM) also made up the highest percentage of HIV diagnoses in 2020 [2]. Prior research has shown a large co-occurrence of rectal bacterial STIs (bSTIs) and HIV acquisition among MSM, which, despite lack of established directionality in much prior research, suggests the use of rectal bSTI screening as a biomedical predictor of future HIV seroconversion [3–5]. Biomedical predictors of HIV incidence, such as detection of rectal *Neisseria gonorrhoeae* (NG) or *Chlamydia trachomatis* (CT) nucleic acids, have also been proposed and used as proxy end points for HIV prevention trials in the era of highly effective pre-exposure prophylaxis (PrEP) [6–8], although more often among studies of women than men [9]. While there has been some research conducted on the ability to predict HIV incidence using rectal bSTIs such as CT or NG, there is need for further research to better establish the magnitude of the association, the directionality of this relationship using longitudinal data, and whether other very common and often asymptomatic rectal bSTIs, like *Mycoplasma genitalium* (MGen), show similar associations.

MGen is an emergent STI of concern in the United States [10]. While often asymptomatic, individuals who test reactive for MGen may experience acute/chronic urethritis and proctitis. Previous research we have conducted has shown a 41.5% prevalence of rectal MGen in SGMs with HIV compared with 16.3% in SGMs without HIV [11]. Cross-sectional studies have also found a strong association between rectal MGen detection and HIV incidence, but there is need to study this relationship using longitudinal data and controlling for behavioral risk as a step toward understanding causation [12]. Further, there has been little epidemiological research examining symptom profiles of rectal bSTIs as most research has been conducted in clinical settings where neither asymptomatic nor self-collected swab rectal bSTI testing is generally conducted. Additionally, while there have been several cross-sectional studies conducted in the past, dynamics, rates, and testing modes continually shift and suggest a need for longitudinal review of this pattern. Thus, the goal of the study was to examine the prospective relationship between molecular detection of multiple rectal bSTI pathogens on self-collected swabs, rectal symptoms, and HIV incidence using data from a longitudinal cohort of YSGMs. Results indicate that detection of NG on self-collected rectal swabs predicts HIV seroconversion after controlling for other risk factors.

## METHODS

Data were collected as part of RADAR, a longitudinal cohort study of YSGMs (n = 1243) living in the Chicago metropolitan area. The primary objective of this cohort study was to apply a multilevel perspective [13] to a syndemic of health issues associated with HIV among diverse YSGMs [14]. Three cohorts of YSGMs (recruited in 2007, 2011, and 2015, respectively) were merged to create a single cohort in this multiple-cohort, longitudinal design [15]. Analogous methods were used to recruit each of the component cohorts, which have been described in more detail elsewhere [16]. At the time of enrollment into their original respective cohorts, all participants were aged 16–20 years, assigned male at birth, spoke English, and had had a sexual encounter with a man in the previous year or identified as gay, bisexual, or transgender. Study visits occurred every 6 months. Rectal swabs were self-collected at each study visit. All procedures were approved by the Northwestern University Institutional Review Board, and participants were compensated for their involvement. For the purposes of these analyses, the analytic sample (n = 1022) consisted of only participants who self-collected swabs for rectal NG, CT, and MGen testing between March 2018 and August 2022. Due to the coronavirus disease 2019 (COVID-19) pandemic, rectal STI testing was not conducted from March 15, 2020, through August 18, 2020, and from November 14, 2020, through March 23, 2021.

### Measures

#### Demographics

Participants reporting Hispanic/Latino ethnicity were coded as such, regardless of their racial identity.

#### Sexual Risk Behavior

The number of condomless anal or vaginal sex partners assigned male at birth in the past 6 months was derived from the HIV Risk Assessment of Sexual Partnerships (H-RASP) [17]. To reduce the impact of extreme values, the number of partners was winsorized at 3 standard deviations from the mean. PrEP use was assessed by asking “Are you currently taking any pre-exposure prophylaxis (PrEP) medication such as Truvada or Descovy to reduce your risk of HIV transmission? (Yes/No)”.

#### HIV Testing

The Alere Determine 4th Generation HIV-1/HIV-2 Ab/Ag Combo rapid test was used to test for the presence of HIV-1 antibodies, HIV-2 antibodies, and free HIV-1 p24 antigen in fingerstick-collected blood at every visit. Laboratory confirmation followed the Centers for Disease Control and Prevention (CDC) guidelines for HIV testing [18]. For each HIV incident case included in these analyses, quantitative polymerase chain reaction (PCR) testing for HIV-1 Gag proteins was conducted using a cryopreserved plasma sample from the visit preceding HIV seroconversion to confirm seronegative status at the prior visit and verify that seroconversion occurred between visits.

#### Rectal STI Testing

The PANTHER system (Hologic, Incorporated, San Diego, CA, USA) was used to test self-collected rectal swab specimens for the presence of CT, NG, and MGen using the Aptima Combo 2 Assay nucleic acid amplification test. Information regarding these molecular assays has previously been published [19].

#### Rectal STI Symptomology

At the time of rectal swab collection, participants were asked to self-report whether they experienced any of the following symptoms in the previous 2 weeks: discharge from rectum, rectal pain or soreness, rectal itching, rectal bleeding, sores or blisters around rectum, and painful bowel movements. The symptoms assessed were selected from the CDC STI fact sheets [20] and were limited to only anorectal symptoms.

### Statistical Analyses

Cross-sectional analyses using each participant's initial study visit post–March 2018 were performed to compute the proportion of rectal MGen, NG, and CT infections and the proportion of coinfections among the sample. Differences in rates of rectal bSTI infection by demographic and behavioral characteristics were not examined as they have previously been published [11]. Next, the proportion of participants reporting rectal bSTI symptoms, stratified by pattern of bSTI results (nonreactive for all bSTI agents, MGen reactive only, NG reactive only, CT reactive only, and reactive for >1 bSTI agent), were calculated, and the Fisher exact test was conducted to test whether significant differences existed. An a priori decision was made to use a Bonferroni-adjusted significance level of .005 when conducting post hoc analyses if Fisher exact testing was significant (*P* < .05). Rectal symptomology questions were not administered at all visits where rectal bSTI testing was conducted, so results represent a subsample (n = 601). Then, using generalized estimating equations (GEEs) with logit link for binary outcomes and an exchangeable working correlation structure, we examined whether the presence of rectal bSTIs predicted HIV seroconversion at the subsequent 6-month follow-up visit. Lastly, we examined this relationship further by including demographic characteristics and sexual risk behavior as covariates. Data collected before a participant's sexual debut, operationally defined as having ever engaged in anal or vaginal sex, were not included in analysis as participants were not considered to be at risk.

## RESULTS

Characteristics of the analytic sample are displayed in Table 1. At the initial study visit post–March 2018, the proportion of participants who tested reactive for rectal MGen, NG, or CT was 20.8% (95% CI, 18.4%–23.5%), 6.5% (95% CI, 5.0%–8.2%), and 8.4% (95% CI, 6.8%–10.3%), respectively; therefore rates of bSTI detection were 3.2 times higher for rectal MGen compared with rectal NG and 2.5 times higher compared with rectal CT. Of the 305 participants who tested reactive for any rectal bSTI, 17.7% were found to be infected with >1 bSTI agent.

**Table 1.** Characteristics of Analytic Sample at Initial Study Visit Post–March 2018 (n = 1022)

	No.	%
Age (mean [SD], 23.4 [3.2])		
16–18 y	40	3.9
19–20 y	237	23.2
21–24 y	424	41.5
25–29 y	292	28.6
30+ y	29	2.8
Gender identity		
Cisgender Man	895	87.6
Transgender Woman	70	6.8
Other	57	5.6
Sexual identity		
Gay	703	68.8
Bisexual	151	14.8
Other	168	16.4
Race/ethnicity		
Black	339	33.2
Latinx	312	30.5
White	258	25.2
Other	113	11.1
HIV positive	204	20.0
PrEP user^a^	143	17.5
CAS partners (median [IQR], 1.0 [2.0])		
0	379	37.1
1	384	37.6
2	110	10.8
3	61	6.0
4 or more	88	8.6

Abbreviations: CAS, condomless anal sex; PrEP, pre-exposure prophylaxis.

^a^Percentage is calculated using only participants who were HIV negative in the denominator (n = 818). Of the 675 participants who were HIV negative and not using PrEP at the initial study visit post–March 2018, 120 (17.8%) started PrEP at a later visit.

Of the participants with a reactive rectal Mgen result and nonreactive rectal NG and CT, the proportion who reported experiencing each of the following rectal bSTI symptoms was as follows: discharge from their rectum (1.3%), rectal pain or soreness (0.0%), rectal itching (1.3%), rectal bleeding (2.5%), sores or blisters around rectum (0.0%), and painful bowel movements (2.5%). A similar pattern of low rectal symptomology was reported by participants who tested nonreactive for all bSTI agents, reactive for NG and CT only, and reactive for >1 bSTI agent. There were no statistically significant differences in prevalence of individual symptoms by pattern of bSTI results (*P* > .05). Of the participants who tested reactive for rectal MGen (n = 79), NG (n = 18), and CT (n = 30), the proportion who reported any rectal bSTI symptom was 6.3% (95% CI, 2.1%–14.2%), 11.1% (95% CI, 1.4%–34.7%), and 10.0% (95% CI, 2.1%–26.5%), respectively. In comparison, of the participants testing nonreactive for all 3 bSTIs (n = 460), the proportion who reported any rectal bSTI symptom was 7.0% (95% CI, 4.8%–9.7%). There were no statistically significant pairwise differences in reported symptoms (asymptomatic vs symptomatic) between participants testing nonreactive vs reactive for rectal MGen (χ^2^ = 0.04; *P* = .84), NG (χ^2^ = 0.45; *P* = .37), or CT (χ^2^ = 0.39; *P* = .46).

Of all participants who were HIV negative at the initial study visit post–March 2018 and had at least 1 follow-up visit, 2.8% seroconverted for HIV. For those participants with reactive rectal MGen, NG, and CT encounters, 1.9%, 4.6%, and 2.0% seroconverted for HIV at the subsequent visit, respectively. In comparison, 0.7%, 0.8%, and 0.9% of all nonreactive encounters seroconverted for HIV at the subsequent visit, respectively. At the visit before HIV seroconversion (n = 18), 55.6% of seroconverting participants had a reactive finding to 1 or more of the rectal bSTI agents. In bivariate GEE analysis, testing reactive for rectal NG (odds ratio [OR], 5.48; 95% CI, 1.53–19.58) or MGen (OR, 3.04; 95% CI, 1.17–7.92) was found to be a statistically significant risk factor for HIV seroconversion compared with participants who tested nonreactive, but the effect was not significant for rectal CT (OR, 2.24; 95% CI, 0.50–10.04). In multivariate analysis, after controlling for demographic characteristics and sexual risk behavior, rectal MGen was not found to be a statistically significant predictor of HIV seroconversion (adjusted odds ratio [AOR], 1.82; 95% CI, 0.61–5.44). However, the presence of rectal NG (AOR, 5.11; 95% CI, 1.20–21.77) and number of condomless anal sex partners (AOR, 1.11; 95% CI, 1.00–1.23) were found to be statistically significant predictors. Results of multivariate GEE analyses are displayed in Table 2. In a supplemental multivariate GEE analysis, including a single binary rectal bSTI predictor instead of 3 individual predictors was found to be significantly associated with HIV seroconversion (AOR, 3.37; 95% CI, 1.22–9.31).

**Table 2.** Generalized Estimating Equations Model Results Predicting HIV Seroconversion (n = 648 Participants, 1806 Observations)

	Estimate	SE	AOR	95% CI	*P* Value
Age	0.12	0.09	1.13	0.94–1.36	.185
Non-Hispanic Black	0.81	0.56	2.24	0.74–6.76	.151
Transgender Woman	−0.29	1.16	0.75	0.08–7.30	.802
PrEP user	0.34	0.53	1.41	0.50–3.95	.527
No. of CAS partners	0.11	0.54	1.11	1.00–1.23	.049
Rectal MGen	0.60	0.56	1.82	0.61–5.44	.286
Rectal NG	1.63	0.74	5.11	1.20–21.77	.027
Rectal CT	0.47	0.91	1.59	0.27–9.49	.610

The sample size for this analysis is smaller than the 1022 participants reported elsewhere because HIV-seroprevalent respondents were excluded from analyses, as well as respondents who did not have data available from a follow-up visit.

Abbreviations: AOR, adjusted odds ratio; CAS, condomless anal sex; CT, *Chlamydia trachomatis*; MGen, *Mycoplasma genitalium*; NG, *Neisseria gonorrhea*.

## DISCUSSION

In this longitudinal sample of YSGMs, there was a large effect size for prediction of HIV seroconversion in individuals who had tested positive for NG on a self-collected rectal swab at a prior visit, regardless of whether the individual had anorectal symptoms, even after adjustment for demographics, 2 other bSTIs, and reported sexual risk behavior. Participants testing NG positive were 5 times more likely to seroconvert by their next visit, which was a larger association than found in prior cross-sectional studies [12]. The total number of condomless anal sex partners (CAS) reported by participants in the last 6 months was also found to be an independent predictor of HIV seroconversion in the multivariate analyses. A similar HIV acquisition risk for testing positive for MGen lost significance after adjustment for demographics, rectal NG, and sexual risks. There has been little research on the biological mechanisms that explain the epidemiological linkage between rectal STIs and future HIV infection risk. One study found that asymptomatic bacterial STIs were associated with rectal inflammation among YMSMs with HIV [21]. More epidemiological and biomedical mechanistic research is needed on this association. This association suggests the value of asymptomatic testing and bSTI treatment for HIV prevention, as well as further research evaluating prioritization of HIV prevention services to YSGMs who test positive for rectal NG, and possibly MGen, on rectal swabs. Routine STI testing regardless of symptoms is recommended for individuals taking PrEP, but perhaps should be expanded to individuals not on PrEP who are at risk for HIV [22].

Detection of bSTI agents was a robust predictor of future HIV seroconversion, with 55.6% of individuals who seroconverted during this period having tested positive for a rectal STI at their prior visit; as noted above, rectal NG detection stood out. A strength of our design was the existing banked samples that allowed us to conduct plasma HIV RNA PCR testing to confirm that HIV seroconversion was subsequent to, rather than contemporaneous with, bSTI detection. Further, controlling for rates of condomless anal sex using a psychometrically validated measure is consistent with our inference that rectal NG detection on self-collected rectal swabs may well have a causal relationship with future HIV seroconversion.

There has been limited prior epidemiology research on MGen in this population. In a study conducting asymptomatic rectal testing in Melbourne, test positivity rates for CT, NG, and MGen were 8.5%, 6.2%, and 7%, respectively. In the current study, participants tested reactive for rectal MGen at 2–3× higher rates than other rectal bSTIs, which is higher than found in other studies [12, 23] but consistent with prior data from this cohort [11]; 17.7% of our participants tested positive for MGen and at least 1 other rectal bSTI, consistent with past findings on MGen [23]. Rates of STI symptoms were low in those testing reactive for rectal MGen, and there were no significant differences in symptom prevalence by MGen status. This was true for other bSTIs as well.

Given the low rate of symptoms in individuals with rectal bSTI detection paired with the high predictiveness of HIV seroconversion after rectal NG detection, our findings support the CDC's current recommendation to conduct asymptomatic testing every 3–6 months in at-risk populations as it increases the likelihood of adequately targeting HIV prevention services to those with the highest likelihood of seroconversion [24]. Currently, there is minimal MGen testing occurring in clinical settings, and it is not currently Food and Drug Administration approved for rectal testing. Further investigation of asymptomatic testing for rectal NG and MGen detection as a means of targeting biobehavioral measures to reduce HIV incidence is necessary to determine if this will make a meaningful difference as there have been trials with mixed results [25]. For example, future research could examine how doxycycline postexposure prophylaxis (DoxyPEP) as a bSTI control strategy may also reduce HIV transmission.

In a 2021 study, it was found that the average estimated lifetime cost of an HIV infection is US$1 079 999 [26]. Thus, the cost of asymptomatic testing in YSGMs could provide cost savings against HIV infection and treatment, as shown in prior modeling studies [6, 27]. It could also reduce bSTI transmission, which is of high importance given that gonorrhea incidence increased yearly from 2017 to 2021, and chlamydia is seeing a resurgence since 2020 [1]. Furthermore, even very expensive HIV interventions that are effective would be cost saving to the health care system when they are directed to individuals at high risk (eg, the CDC estimates that interventions costing up to $402 000 in 2011 $US result in cost savings), especially considering the lifelong treatment needed for HIV infection acquired by youth [28]. Given the very strong predictive relationship found here, it is likely that even expensive HIV prevention interventions would be cost saving when delivered to YSGMs who are HIV negative and test positive for rectal NG and perhaps MGen. With highly effective biobehavioral prevention interventions already established, future studies should assess the efficacy as well as what kinds of adjunctive interventions (eg, addressing clients’ motivational and structural barriers to utilization) and implementation strategies (eg, health system changes) maximize this HIV prevention opportunity [29, 30].

Our study is not without its limitations. Our data are derived from a community sample rather than a probability sample, so there is a possibility that there are sections of the target population that are not represented in these data. Additionally, the COVID-19 pandemic required gaps in the collection of rectal STI samples for 9 months of our study period, 2018–2022. It is also likely that participants’ sexual and/or service-seeking behaviors changed during those periods as they were at peak times of new COVID-19 strains. Therefore, it is possible that there were novel patterns that came out of that period that were not captured. Finally, PrEP use was not significantly associated with HIV seroconversion, despite its well-documented efficacy during adherent use. In fact, our odds ratio was in the opposite direction of protection (1.41), but based on the 95% confidence interval (0.50–3.95) we cannot unequivocally state that PrEP use during a prior wave was a risk factor for future HIV seroconversion. We hypothesize several reasons for our results. First, current PrEP use was included in the model and measured at the visit before HIV seroconversion, so it is possible that participants discontinued use before HIV acquisition and that is not being captured in our data. In fact, our own research in this cohort, and several other studies based on clinical and insurance data, shows a high rate of PrEP discontinuation over a 6-month period [31–34]. Furthermore, our own published research in this cohort shows that HIV transmission risk behaviors continue after PrEP discontinuation, marking discontinuation as a period of high risk for transmission [35]. Second, and similarly, adherence to PrEP medication at the time of seroconversion is not being captured in this model and is something that could explain this finding.

Prior research has shown that HIV seroconversion may no longer be effectively used as an end point in future trials of novel PrEP agents given that existing highly effective PrEP prevents ethical studies from offering a placebo and that rectal NG could serve as a proxy end point [25]. In our study, individuals testing positive for rectal NG had the highest risk of seroconversion, which is in line with prior findings. Our research therefore provides a more robust estimation of the relationship between NG and HIV among YSGMs that could be used as a parameter in modeling effects using their proposed approach.
